# Carbon monoxide poisoning-induced cardiomyopathy from charcoal at a barbecue restaurant: a case report

**DOI:** 10.1186/s40557-015-0063-2

**Published:** 2015-04-28

**Authors:** Hyun-Jun Kim, Yun Kyung Chung, Kyeong Min Kwak, Se-Jin Ahn, Yong-Hyun Kim, Young-Su Ju, Young-Jun Kwon, Eun-A Kim

**Affiliations:** Department of Occupational and Environmental Medicine, Hallym University Sacred Heart Hospital, Anyang City, Republic of Korea; Center for Occupational Health Research, Occupational Safety and Health Research Institute, Korea Occupational Safety and Health Agency, Ulsan City, Republic of Korea

**Keywords:** Carbon monoxide poisoning, Cardiomyopathy, Charcoal, Restaurant worker

## Abstract

**Objective:**

Acute carbon monoxide poisoning has important clinical value because it can cause severe adverse cardiovascular effects and sudden death. Acute carbon monoxide poisoning due to charcoal is well reported worldwide, and increased use of charcoal in the restaurant industry raises concern for an increase in occupational health problems. We present a case of carbon monoxide poisoning induced cardiomyopathy in a 47-year-old restaurant worker.

**Materials and methods:**

A male patient was brought to the emergency department to syncope and complained of left chest pain. Cardiac angiography and electrocardiography were performed to rule out acute ischemic heart disease, and cardiac markers were checked. After relief of the symptoms and stabilization of the cardiac markers, the patient was discharged without any complications.

**Results:**

Electrocardiography was normal, but cardiac angiography showed up to a 40% midsegmental stenosis of the right coronary artery with thrombotic plaque. The level of cardiac markers was elevated at least 5 to 10 times higher than the normal value, and the carboxyhemoglobin concentration was 35% measured at one hour after syncope. Following the diagnosis of acute carbon monoxide poisoning induced cardiomyopathy, the patient’s medical history and work exposure history were examined. He was found to have been exposed to burning charcoal constantly during his work hours.

**Conclusions:**

Severe exposure to carbon monoxide was evident in the patient because of high carboxyhemoglobin concentration and highly elevated cardiac enzymes. We concluded that this exposure led to subsequent cardiac injury. He was diagnosed with acute carbon monoxide poisoning-induced cardiomyopathy due to an unsafe working environment. According to the results, the risk of exposure to noxious chemicals such as carbon monoxide by workers in the food service industry is potentially high, and workers in this sector should be educated and monitored by the occupational health service to prevent adverse effects.

## Background

Acute carbon monoxide poisoning is a clinically imperative condition since the poisoning can lead to severe cardiovascular complications and/or sudden death [1]. Cardiac problems associated with carbon monoxide poisoning are usually described as delayed myocardial injury [2] or carbon monoxide induced cardiac damage [3], but no clinical term has ever been defined for the cardiac injury induced by carbon monoxide poisoning. There have been several reports about healthy individuals suffering from the same injury after work related exposures to carbon monoxide [4]. Thus, in addition to accidental exposures, carbon monoxide poisoning is now also believed to result from occupational exposures among workers’ who are directly and chronically exposed to a high amount of the gas.

Generally, in an open environment, carbon monoxide is generated by the incomplete combustion of fuel sources such as wood, charcoal, oil, paraffin, propane, natural gas, and waste or exhaust gas emitted by car engines, portable power generators, lawn mowers, and washers. In addition, smoke and heat can lead to an additional carbon monoxide deposition especially in closed spaces [5].

Carbon monoxide poisoning resulting from burning charcoal has been frequently reported in Asian countries including South Korea [6]. In Europe and the United States, using a charcoal barbecue grill is considered hazardous due to the potentially hazardous levels of carbon monoxide produced in these grills [7-9]. In Australia, one case died of carbon monoxide poisoning after using charcoal for cooking purposes [10].

Recently, the incidence of carbon monoxide poisoning from coal heating has decreased dramatically in South Korea, but the use of charcoal and coal in the service industry has substantially risen. Workers in the service industry are usually considered at much lower risk of exposure to physical and chemical hazardous materials than those working in the manufacturing industry are, and this perception has led to less rigid occupational safety and health regulations. However, without a proper understanding about the occupational safety and health of workers in South Korea, the increased proportion of workers in the service industry at risk of exposure to carbon monoxide and the substantial increase in the amount of charcoal or coal use in this industry has led to a potentially dangerous level of exposure among these workers.

Therefore, we studied a patient who experienced cardiomyopathy after carbon monoxide poisoning and investigated the degree of carbon monoxide exposure in the work place through an occupational epidemiological survey. Any potential material that might have been associated with the patient’s condition was studied and the association between the incidence of the disease and the patient’s work was evaluated. Moreover, we observed any changes in the patient’s clinical progress after quitting his job. Last, our aim was to investigate possible guidelines for assessing work-related factors in this case toward the future progress in obtaining proper and timely diagnoses of carbon monoxide induced cardiomyopathy. Moreover, this report aims to raise awareness about occupational hazards among workers in the food service industry who are exposed to daily charcoal use.

## Case report

**Name:** Mr. Choi

**Gender and age:** Male, 47 years old

**Chief complaint:** Left chest pain

## Case presentation

The patient started working for a Korean-style barbecue restaurant from January 5, 2013. His primary duty was to ignite and manage the charcoal used for barbecuing meat at the restaurant. He worked 10 hours a day six days a week. On January 19, 2013, 14 days after he had started working, he suddenly collapsed and lost consciousness while on duty. The length of period for which he lost of consciousness is unknown; however, he was observed as alert and working approximately 30 to 40 minutes prior to the syncope. He regained consciousness without any medical treatment, and no medical attention was provided. However, he complained of a squeezing pain in his left chest for one hour after the initial episode; thereafter, he was brought to hospital via ambulance. Within one hour of arriving at the emergency room, he received cardiac angiography and was admitted to the hospital. Diagnosis at the time of admission was unstable angina and carbon monoxide poisoning. In the emergency room, his electrocardiogram was normal, and his systolic and diastolic blood pressures were 125 mmHg and 74 mmHg, respectively. He was discharged without any complications. On January 31, 2013, he filed a claim with the Korea Worker’s Compensation and Welfare Service seeking compensation for the cardiac incidence that was believed to have been related to his work managing the charcoal at the restaurant. Before a decision regarding his claim could be made, the service requested an occupational epidemiological study from the Occupational Safety and Health Research Institute, and our hospital’s occupational and environmental medicine department was given the task of creating the epidemiological report. After further investigation by our department, it was concluded that the patient had carbon monoxide induced cardiomyopathy after exposure to hazardous materials in his work place. The informed consent for epidemiologic investigation and case report is performed.

### Past medical and personal history

In August 2012, he had been diagnosed with nonspecific hypertension (Korean standard classification of diseases code I10.9) at Gangdong district health center, but we were unable to find his exact blood pressure measurement at the time of this diagnosis. The patient testified that he was not taking hypertension medication. His medical history was otherwise insignificant. Prior to January 2013, he had visited a local clinic intermittently seeking treatment for shoulder and back pain. According to his medical records until January 19, 2013, the patient had no remarkable family or social history and had never been exposed to any radiologic treatment. However, we were not able to confirm any of these records using the patient’s annual medical check-up, which is managed by the National Health Insurance Service. He told the medical personnel at our hospital that he smokes 10–20 cigarettes per day, but his medical record from Samsung Medical Center showed a record of 20 pack-years. He also reported rarely consuming alcohol.

### Physical examination

At the emergency room, his systolic and diastolic blood pressures were 125 mmHg and 74 mmHg, respectively, with a pulse rate of 65 beats per minute, a respiratory rate of 18 breaths per minute, and a body temperature of 36°C. Upon auscultation, a rale was heard in both lung fields. Otherwise, no abnormal findings were reported.

### Laboratory examination

The results of the electrocardiogram taken at admission were normal. Table 1 lists the initial and follow-up concentrations of carboxyhemoglobin, methemoglobin, and hemoglobin oxygen saturation. The carboxyhemoglobin concentration at the emergency room was 35.8%, which is significantly higher than the normal level. In addition, the results of the cardiac enzyme tests are shown in Table 2. Troponin I, which typically indicates cardiac injury, was considerably elevated.

**Tables 1:** **The laboratory datas for diagnosis of mono-carbon intoxication**

**Laboratory indices**	**Normal value (%)**	**2013.1.19 13:51**	**2013.1.19 16:22**	**2013.1.19 21:55**	**2013.1.20 04:38**	**2013.1.20 11:25**
CO Hb	0.5-1.5	35.8	11.9	1.7	1.6	1.4
Methemoglobin	0.4-1.5	0.3	0.3	0.1	0.3	0.3
O2 Hb	94-97	17.8	87.1	97.7	97.4	97.4
Total Hb	12-18*	15.6	13.7	12.8	13.1	13.9

*Unit: g/dL.

**Table 2:** **The laboratory datas for diagnosis of cardiomayopathy**

**Laboratory indices**	**Normal value (ng/dl)**	**2013.1.19 14:08**	**2013.1.19 21:07**	**2013.1.20 04:51**	**2013.1.20 11:40**	**2013.1.20 22:30**
Troponin I	0–0.78	0.01	6.15	7.30	4.03	2.06
Myogloblin	0–110	44.87	-	-	-	-
CK-MB	0–5	1.54	18.69	20.54	14.21	5.45

### Results of the cardiac angiography

A cardiac angiogram was performed within one hour of admission to the emergency room. In the right coronary artery, a 40% obstruction and thrombotic plaque were found, but the other three branches of the coronary artery appeared normal.

### Results of the cardiac contrast ventriculography

A cardiac contrast ventriculography was performed, and the myocardium and cardiac output were found to be functioning normally.

### Clinical progress

Initially, the patient’s systolic and diastolic blood pressures were 125 mmHg and 74 mmHg, respectively. His pulse and respiratory rates were 65 beats per minute and 18 breaths per minute, respectively, and he had an oxygen saturation of 98% at room temperature. He was given emergency therapy with a sublingual nitroglycerine. After taking the medication, the only response was a sudden drop in his systolic and diastolic blood pressures to 83 mmHg and 53 mmHg, respectively. To counter this low blood pressure, 500 mL of normal saline was infused intravenously, and after confirming the stabilization of his vital signs, the patient underwent an emergency angiocardiogram. He was admitted to the intensive care unit for close observation immediately after the procedure. An angiotensin converting enzyme inhibitor (Losartan®) and aspirin were prescribed, and his systolic and diastolic blood pressures remained level at approximately 135 mmHg and 80 mmHg, respectively. He was discharged from the hospital on January 22, 2013, four days after his first visit to the emergency department.

After discharge, he did not return to his previous vocation. He visited our outpatient clinic on January 31, 2013, 14 days after the initial attack. At that time, his systolic and diastolic blood pressures were 116 mmHg and 79 mmHg, respectively, and he reported no longer suffering from chest pain or other cardiac symptoms. The angiotensin converting enzyme inhibitor and aspirin were prescribed for continuous use. His last visit was on February 21, 2013 to obtain a copy of his medical record. At the last visit, no chest pain was present, and his systolic and diastolic blood pressures were 143 mmHg and 100 mmHg, respectively.

### Occupational history and environment

According to this patient’s insurance employment records, his first full-time recorded job was at a Korean barbecue grill restaurant. He started working there on January 5, 2013 (at 46 years old), and prior to this full-time employment, he worked as a part time employee from June 2, 2012 to August 1, 2012 cleaning tables and washing dishes.

**Figure 1:**
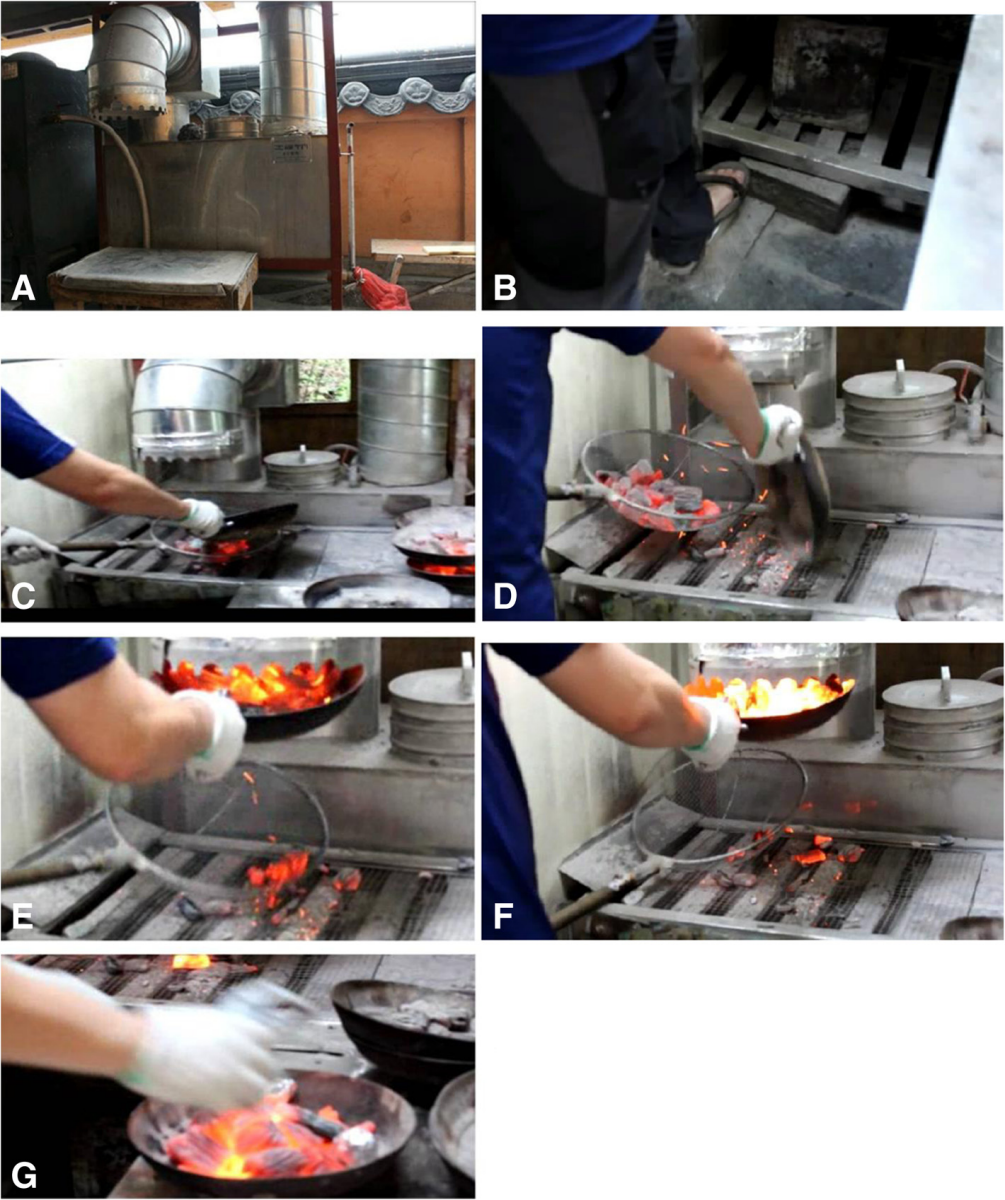
The occupational processes and tools at workplace. **A**. The charcoal recycler. **B**. The on/off button of the charcoal recycler. **C**. Handling the charcoal bowl. **D**. Moving the charcoal to the grid pan. **E**. After a fire is started in the charcoal with the grid pan, the burning charcoal is moved from the pan to the bowl. **F**. Placing and stirring the burning charcoal beneath the duct for aeration. **G**. Moving the charcoal bowl to a metal container for delivery to tables.

At Korean barbecue grill restaurant, he worked at the charcoal recycler (Figure 1), which was located outside of the restaurant, and his job was to ignite fresh charcoal or revive the flames of burning charcoal to be placed on customers’ tables in the restaurant. His informal duties included washing charred material from gridirons and moving stacks of charcoal for storage. He washed used gridirons once or twice a day, usually in the morning or late at night. His job was also to remove large deposits of soot, first using a device called a ‘grand’, and then using a metallic brush to scrape off any charred materials and minor soot from the gridiron. He performed these tasks outside of the restaurant. It is unknown whether the patient also used chemical agents while cleaning. An exhaust duct is fitted above the recycler. Smoke from the fire at the charcoal recycler is cleared by natural air ventilation, and no additional circulation is provided to ventilate the unwanted smoke.

He worked for six days a week, from 10 AM to 10 PM. He was given an hour and a half lunch break. The quantity of charcoal he managed each day and the amount of his daily workload depended on the number of patrons who visited the restaurant that day. When the restaurant was not busy, he had anywhere between several minutes to one hour of break time throughout his day, and he usually rested next to the charcoal recycler. The estimated amount of charcoal used per day was three to four boxes (6–10 kg). We requested an estimate of the number of customers and daily sales figures from the business owner in an attempt to estimate the quantity of charcoal used per day. However, even with this information, the amount of charcoal handled by the patient or used at a table in this restaurant is difficult to estimate because it can vary greatly for each table. Charcoal is provided at customer’s request or after a further order of meat.

The steps to operating the charcoal recycler, which was used by the patient, are as follows. There is on and off button on the bottom of the recycler. After turning on the recycler, the appropriate amount of fresh charcoal for single use is loaded into the charcoal bowl and moved from the bowl to the grid pan. Then, the pan is placed below the ventilation duct in the center of the recycler. With aeration, a flame is applied to start a fire in the charcoal. The flaming charcoal is then placed in a bowl, and the bowl is then placed just under the duct for more aeration. When the charcoal becomes hot enough for grilling, the burning charcoal is moved to another metal container for the use at a customer’s table. The whole procedure takes 30 to 40 seconds.

## Discussion and conclusions

According to Korea Occupational Safety and Health Agency’s data from the year 2011, a total of 29,736 workers in the service industry suffered an occupation-related injury. These workers account for 32% of all occupation associated injuries or illnesses in South Korea. Among them, the hotel and restaurant sector had 6,995 workers who had suffered from occupational accidents. This sector is one of the areas that seem to be neglected, and researchers in the field of occupational and environmental medicine have expressed interest in overseeing and managing preventive health strategies among these workers. However, even with the recent boom in certain service industries and the increased number of workers, a systematic approach to improving the safety and health of these workers remains a great challenge.

Herein, we reported on our experience with a worker from the restaurant industry who became intoxicated with carbon monoxide while working. The intoxication eventually led to cardiomyopathy, a cardiac complication, in the patient. We reported on this particular case and described of the clinical characteristics and progress of treating this patient. Furthermore, we have made an effort to highlight the dangers of charcoal handling and provide information on its adverse effects to other workers in the restaurant industry.

### Evaluation of the working environment

After evaluating the patient’s working environment, it became clear that the patient was exposed to carbon monoxide from the incomplete combustion of charcoal while moving the charcoal from the bowl to the netted pan and back to the bowl in the charcoal recycler. In addition, when the charcoal is placed under the duct for aeration and stirred to move the flame around the charcoal evenly, the pan would then be closer to patient’s nose and mouth, increasing the likelihood of carbon monoxide inhalation. We concluded that the patient’s posture and position in relation to the pan determined the amount of carbon monoxide he inhaled due to the incomplete combustion of charcoal. Finally, the burning charcoal was placed in a separate metal container for delivery to each table, and on a busy day, a greater number of containers with burning charcoal were prepared and placed next to the recycler. The presence of additional containers likely increased his exposure to carbon monoxide.

A recent report stated that a carbon monoxide exposure of greater than 1200 ppm causes immediate danger to life and/or long-term health [11]. In addition, a laboratory test involving rats reported that a carbon monoxide concentration of 50 ppm was lethal and that poisoning resulted after an exposure to 1807 ppm over 4 hours.

In a field study in Great Britain that was performed in restaurants using charcoal for grilling, the amount of carbon monoxide generated was measured, and according to their data, the carbon monoxide emission next to a container with burning charcoal was 150 ppm to 500 ppm [12]. A similar study was performed in Japan where carbon monoxide emissions from burning charcoal was 137 mL/(min · kW)^−1^ to 185 mL/(min · kW)^−1^, and this range is thought to be substantially higher than the amount of carbon monoxide that can be cleared by natural ventilation [13].

Therefore, although the recycler was placed outside with a natural ventilation duct attached, we believe that the patient was still exposed to a substantial amount of carbon monoxide gas while working at the charcoal recycler.

### Medical considerations

Health issues related to charbroiling or grilling over charcoal include carbon monoxide intoxication from exposure to the incomplete combustion of the carbon source [14]. In addition, exposure to polynuclear aromatic hydrocarbons and the phenol and creosote in burning beech wood as well as the inhalation of these substances is of concern because of the potential carcinogenic effects these substances can have on the nose and skin [15].

The clinical symptoms of carbon monoxide poisoning can vary by the length and quantity of the exposure; nonspecific symptoms arise from the formation of carboxyhemoglobin, ischemic changes in the tissue, and carbon monoxide directed cell damage [16]. The most notable symptoms include headache, nausea, vomiting, confusion, fatigue, chest pain, dyspnea, and loss of consciousness [17]. Other symptoms that are associated with carbon monoxide intoxication in cold weather are similar to the symptoms associated with acute coronary disease and arrhythmia [18].

The health related consequences of carbon monoxide poisoning vary as wide as its symptoms. The most susceptible organs are the basal ganglia, white matter, and cerebral cortex of brain and heart, all of which demand a substantially greater amount of oxygen than other organs do [19]. In addition, acute carbon monoxide intoxication increases the prevalence of micromolecules that originate in the blood, and it leads to extravasation and the dissociation of neutrophils. In turn, these macromolecules cause injuries and impair the functions of the vascular and central nervous systems [20]. Symptoms of acute carbon monoxide intoxication occur immediately after the exposure, but nausea, chest pain and hearing difficulties can be develop as sequelaes. Severe neuropsychiatric complications may occur two to 28 days after the acute intoxication [19].

In the cardiovascular system, the myocardium is especially sensitive to oxygen saturation, and increased contraction strength, reduced coronary arterial circulation, and increased oxygen demand in the cardiac myocytes further aggravate the oxygen deficiency, thus worsening the injury. Recently, Lippiet al. [21] showed that the right atrium is more likely to be damaged from an intoxication of carbon monoxide. In another study, approximately 30% of patients had abnormal electrocardiogram findings, and elevated levels of cardiac enzymes were evident in 35% of these patients [22]. A reduced ejection fraction in the left atrium and an increased level of brain natriuretic protein were also observed [23]. Carbon monoxide’s affinity for oxygen is 60 times higher than that of hemoglobin’s is, and this higher affinity prevents oxygen from binding to the mitochondria. The amount of energy supplied to the myocardium directly causes mitochondrial damage and decreases the vicious effects on the myocardium. At the same time, myocardial fibrosis and cardiomyopathy arise from the injury. Finally, injury leads to a decreased function of the left atrium, further diminishing circulation to the heart [21].

Electrocardiography, measurements of cardiac enzymes, imaging studies such as echocardiography, and cardiac angiography are recommended to confirm the diagnosis of a cardiomyopathy induced by carbon monoxide poisoning. On electrocardiogram, inversion or loss of the T wave and ST depression or elevation suggestive of myocardial infarction may be present, and imaging studies such as a cardiac magnetic resonance imaging and Single photon emission computed tomography should be performed to confirm the diagnosis [21]. Cardiac enzymes including creatine kinase, creatine kinase MB fraction, and troponin I may also assist in making the diagnosis. In our case, the patient’s CK-MB and troponin I were elevated, indicating cardiac injury induced by carbon monoxide intoxication. By measuring blood carboxyhemoglobin concentration, it is possible to infer the amount of the exposure. For example, if the concentration is 30%, the atmospheric carbon monoxide concentration is estimated to be 220 ppm. For a carboxyhemoglobin concentration between 40% and 50%, the estimated concentration of the gas is estimated to be between 350 ppm and 520 ppm [19]. Thus, our patient with a blood carboxyhemoglobin concentration of 35% measured at one hour after the initial chest attack is thought to have been exposed to more than 220 ppm of carbon monoxide.

### Relationship between the occupational environment and the accident and injury

The patient experienced carbon monoxide intoxication evidenced by a 10 times higher concentration of carboxyhemoglobin than normal measured immediately after the episode. Since his work required the manual handling of charcoal, we also believe that prior to the attack the patient was chronically exposed to a considerably high amount of the gas. The exposure time precedes the point of diagnosis, and the biological consequence found in the patient was similar to the complications found in the hazard. The patient reported no recurrence or aggravation of these symptoms once he quit his job after being released from the hospital.

Prior to the illness, the patient had a history of hypertension, but a history of hypertension is generally not considered as a risk factor of cardiomyopathy induced by carbon monoxide intoxication. In addition, on cardiac angiogram, a rupture of a thrombotic plaque in the right coronary artery of the heart was found and is considered an obstruction that can result in the rapid onset of atherosclerotic changes in the heart. Carbon monoxide often creates a thrombus, and hypoxia from carbon monoxide intoxication is known to work as a stimulus that incites the plaque to rupture [24].

The patient worked for 14 days at the barbeque grill restaurant from January 5 to 19, 2013, and his primary duty at the job was to handle charcoal that is served to the table for grilling. The patient suffered acute carbon monoxide poisoning while working at the restaurant. The level of blood carboxyhemoglobin concentration measured one hour after the attack was 35%. The extent of myocardial injury is not clear from the findings of the electrocardiography and cardiac angiography. Confirming the diagnosis of the intoxication with elevation of cardiac enzymes and evidence of exposure to significant amounts of carbon monoxide suggest a considerable association and possibility that these factors influenced the development of the cardiovascular complication. Thus, in our patient, the incidence of cardiac injury is clearly and strongly associated with his occupation.

To the best of our knowledge, this is the first case reported in South Korea that concerns a worker from the restaurant sector of the service industry who handled charcoal and suffered from carbon monoxide poisoning with a complication of cardiomyopathy. However, there are some limitations in this case report. We did not perform an onsite measurement of the carbon monoxide exposure at his work place, and we were not able to follow the patient long term for any potential complications that might arise.

Despite these weaknesses, we believe that this report can provide some basis for the future evaluation of workers in the restaurant sector or similar sectors who use charcoal for grilling to inform them about the occupational relationship between carbon monoxide poisoning and cardiovascular diseases. Our case might be an index case that screening and health exam related evaluation procedures for workers with cardiovascular diseases should be performed before assigning them to particular services such as charbroiler, which might be fatal to their health. For physicians, when they encounter a patient with nonspecific clinical or imaging findings of the cardiovascular system and that patient works in/around grills, they should presume that the patient might have been substantially exposed to carbon monoxide. Moreover, examinations to rule out carbon monoxide poisoning must be performed together with other clinical tests to find the exact cause of the nonspecific findings. In addition, when patients with cardiovascular disease are diagnosed with precedent carbon monoxide poisoning, changes in their clinical progress must be followed closely even after the patient stops working around the source of carbon monoxide exposure.

Lastly, it is imperative to educate both business owners and workers about the danger of acute carbon monoxide poisoning. To prevent the intoxication, an appropriate ventilator system should be installed, and workers should be educated on how to avoid the inhalation of the hazardous gas. Since the potential hazardous effect of carbon monoxide poisoning in this type of work place has been overlooked by the conventional occupational health service industry, basic education to raise awareness about the dangers of this exposure should be initiated immediately. A poor understanding among workers about this risk means that they are likely to stay within an area of high carbon monoxide concentration even during their break time. Therefore, a separate location for resting during breaks should be provided to prevent unnecessary exposure to hazardous materials. Our report not only reported on a rare case of carbon monoxide poisoning in a worker whose vocation has been previously overlooked by the mainstream occupational health service but has also raised awareness for an important issue about the necessary, continuous monitoring and systematic management of occupational health in workers in the service industry.
